# Supplementary material for: The Era of DAAs: Assessing the Patients’ Characteristics, Clinical Impact, and Emergence of Comorbidities in HIV/HCV-Coinfected versus HIV-Infected Individuals
Source: J Clin Med. 2024 Jul 4;13(13):3936. doi: 10.3390/jcm13133936 (PMC11242478; doi:10.3390/jcm13133936)
Supplement: Supplementary file 1 [file jcm-13-03936-s001.zip › jcm-3055217-supplementary.pdf]

TITLE: The Era of DAAs: Assessing the Clinical Impact and Emergence of Comorbidities in HIV/HCV-Coinfected versus HIV-Infected Individuals

AUTHORS: Álvarez-Álvarez, Beatriz [1]*; Prieto-Pérez, Laura[1]; de la Cuadra-Grande, Alberto [2]; Casado, Miguel Ángel [2]; Cabello Úbeda, Alfonso [1]; Al-Hayani, Aws W.[1]; Carrillo Acosta, Irene [1]; Mahillo-Fernández, Ignacio [3]; Górgolas Hernández-Mora, Miguel [1]; Benito, Jose M. [4, 5]; Rallón, Norma [4, 5].

[1] Division of Infectious Diseases. Hospital Universitario Fundación Jiménez Díaz.

[2] Pharmacoeconomics & Outcomes Research Iberia (PORIB). Paseo Joaquín Rodrigo 4, Letter I, Pozuelo de Alarcón, 28224, Comunidad de Madrid, Spain.

[3] Biostatistics and Epidemiology Unit. Instituto de Investigación Sanitaria-Hospital Universitario Fundación Jiménez Díaz, Universidad Autónoma de Madrid (IIS-FJD, UAM), Madrid, Spain.

[4] HIV and Viral Hepatitis Research Laboratory, Instituto de Investigación Sanitaria Fundación Jiménez Díaz, Universidad Autónoma de Madrid (IIS-FJD, UAM), Madrid, Spain.

[5] Hospital Universitario Rey Juan Carlos, Móstoles, Spain.

* Correspondance: balvarez@quironsalud.es

SUPPLEMENTARY FILES

Table S1. Additional demographics characteristics of the patients: AIDS-conditions.

		HIV/HCV Group (N = 229)	HIV Group (N = 229)	p-Value
**Tuberculosis**				0.621 [1]
No	N (%)	219 (95.6%)	222 (96.9%)	
Yes	N (%)	10 (4.4%)	7 (3.1%)	
**Recurrent pneumonia**				0.013 [1]
No	N (%)	221 (96.5%)	229 (100%)	
Yes	N (%)	8 (3.5%)	0 (0.0%)	
***Pneumocystis* pneumonia**				0.786 [1]
No	N (%)	223 (97.4%)	221 (96.5%)	
Yes	N (%)	6 (2.6%)	8 (3.5%)	
**Esophageal candidiasis**				1.000 [1]
No	N (%)	220 (96.1%)	220 (96.1%)	
Yes	N (%)	9 (3.9%)	9 (3.9%)	
**Kaposi's sarcoma**				0.275 [1]
No	N (%)	215 (93.9%)	221 (96.1%)	
Yes	N (%)	14 (6.1%)	8 (3.5%)	
**Cryptococcosis**				0.132 [1]
No	N (%)	225 (98.3%)	229 (100%)	
Yes	N (%)	4 (1.7%)	0 (0.0%)	
**Cerebral toxoplasmosis**				0.368 [1]
No	N (%)	225 (98.3%)	228 (99.6%)	
Yes	N (%)	4 (1.7%)	1 (0.4%)	
**Non-Hodgkin lymphoma**				1.000 [1]
No	N (%)	224 (97.8%)	225 (98.3%)	
Yes	N (%)	5 (2.2%)	4 (1.7%)	
**Progressive multifocal leuco-encephalopathy**				1.000 [1]
No	N (%)	229 (100%)	228 (99.6%)	
Yes	N (%)	0 (0.0%)	1 (0.4%)	

AIDS: Acquired Immunodeficiency Syndrome; HCV: Hepatitis C Virus; HIV: Human Immunodeficiency Virus.

[1] Differences between the HIV/HCV and HIV groups according to the chi-square test or Fisher's exact test.

Table S2. Additional demographics characteristics of the patients: Specific-drug use.

		HIV/HCV Group (N = 229)	HIV Group (N = 229)	p-Value
**Slam consumption**				< 0.001 [1]
No	N (%)	177 (79.0%)	228 (99.6%)	
Yes	N (%)	47 (21.0%)	1 (0.4%)	
**Inhaled cocaine consumption**				< 0.001 [1]
No	N (%)	112 (53.1%)	213 (93.0%)	
Yes	N (%)	99 (46.9%)	16 (7.0%)	
**Parental cocaine consumption**				< 0,001 [1]
No	N (%)	170 (80.6%)	228 (99.6%)	
Yes	N (%)	41 (19.4%)	1 (0.4%)	
**GHB consumption**				< 0.001 [1]
No	N (%)	145 (68.7%)	220 (96.1%)	
Yes	N (%)	66 (31.3%)	9 (3.9%)	
**Ketamine consumption**				0.202 [1]
No	N (%)	202 (95.7%)	225 (98.3%)	
Yes	N (%)	9 (4.3%)	4 (1.7%)	
**MDMA consumption**				0.083 [1]
No	N (%)	203 (96.2%)	227 (99.1%)	
Yes	N (%)	8 (3.8%)	2 (0.9%)	
**Inhaled mephedrone consumption**				< 0.001 [1]
No	N (%)	131 (62.1%)	218 (95.2%)	
Yes	N (%)	80 (37.9%)	11 (4.8%)	
**Parental mephedrone consumption**				< 0.001 [1]
No	N (%)	162 (76.8%)	227 (99.1%)	
Yes	N (%)	49 (23.2%)	2 (0.9%)	
**Methamphetamine consumption**				< 0.001 [1]
No	N (%)	174 (82.5%)	225 (98.3%)	
Yes	N (%)	37 (17.5%)	4 (1.7%)	
**Speed/Amphetamine consumption**				< 0.001 [1]
No	N (%)	196 (92.9%)	229 (100%)	
Yes	N (%)	15 (7.1%)	0 (0.0%)	
**Popper consumption**				< 0.001 [1]
No	N (%)	144 (68.2%)	223 (97.4%)	

Yes	N (%)	67 (31.8%)	6 (2.6%)	
**Sildenafil consumption**				< 0.001 [1]
No	N (%)	172 (81.5%)	227 (99.1%)	
Yes	N (%)	39 (18.5%)	2 (0.9%)	
**Heroine consumption**				< 0.001 [1]
No	N (%)	162 (76.8%)	227 (99.1%)	
Yes	N (%)	49 (23.2%)	2 (0.9%)	

HCV: Hepatitis C Virus; HIV: Human Immunodeficiency Virus.

[1] Differences between the HIV/HCV and HIV groups according to the chi-square test or Fisher's exact test.
